# CDC Grand Rounds: The Growing Threat of Multidrug-Resistant Gonorrhea

**Published:** 2013-02-15

**Authors:** Edward W. Hook, William Shafer, Carolyn Deal, Robert D. Kirkcaldy, John Iskander

**Affiliations:** Univ of Alabama, Birmingham; Emory Univ, Atlanta, Georgia; National Institute for Allergy and Infectious Disease, National Institutes of Health, Bethesda, Maryland; Div of STD Prevention, National Center for HIV/AIDS, Viral Hepatitis, STD, and TB Prevention; Office of the Associate Director for Science, CDC

Although gonorrhea has afflicted humans for centuries, and the causative bacterium, *Neisseria gonorrhoeae*, was identified more than a century ago, gonorrhea remains a public health problem in the United States. Gonorrhea is the second most commonly reported notifiable infection in the United States; >300,000 cases were reported in 2011 (1). In the United States, health inequities persist; the incidence of reported gonorrhea among blacks is 17 times the rate among whites, likely because of structural socioeconomic factors (1,2).

Infection with *N. gonorrhoeae* is spread through sexual contact and, depending on the anatomic site of exposure, can cause acute urethritis, cervicitis, proctitis, or pharyngitis. However, most cases of gonorrhea are asymptomatic, particularly cervical, pharyngeal, and rectal infections. Untreated or inadequately treated gonorrhea can facilitate human immunodeficiency virus (HIV) transmission and cause serious reproductive complications in women, such as pelvic inflammatory disease, ectopic pregnancy, and infertility. Other severe complications, including disseminated gonococcal infection and neonatal conjunctivitis and blindness, still occur in resource-limited settings, but are now rare in the United States. Empiric antimicrobial therapy is used for treatment of gonorrhea. Antimicrobial susceptibility testing generally is not routinely available in clinical practice, and early diagnosis and effective antimicrobial treatment of patients and their partners has been the mainstay of gonorrhea control and prevention; thus, gonococcal antimicrobial resistance poses a grave challenge.

Before the 1930s, gonorrhea often was treated with patent medicines or intraurethral irrigations with compounds such as merbromin (Mercurochrome) or other antiseptics. The introduction of sulfonamide antimicrobials in the 1930s ushered in an era of effective antimicrobial therapy for gonorrhea. However, widespread gonococcal resistance to sulfonamides occurred rapidly and was common by the 1940s. Penicillin was then found to be effective for gonorrhea treatment and became the therapy of choice for several decades. During this time, however, the gonococcus acquired genetic mutations that conferred increasing penicillin resistance, necessitating increasingly higher doses of penicillin to ensure treatment success. By 1976, through further mutations, the gonococcus became able to produce beta-lactamase, an enzyme that destroys penicillin; strains that produce this enzyme are highly resistant to penicillin. During the 1980s, penicillin- and tetracycline-resistant strains became widespread in the United States, complicating gonorrhea therapy.

Recognizing the need for ongoing surveillance of gonococcal antimicrobial resistance, CDC developed the Gonococcal Isolate Surveillance System (GISP) in 1986. GISP is a CDC-supported sentinel surveillance system that monitors gonococcal antimicrobial susceptibility among urethral *N. gonorrhoeae* isolates collected from men attending participating sexually transmitted disease (STD) clinics.* The objectives are to provide a scientific basis for gonorrhea treatment recommendations and to allow changes in treatment recommendations before widespread treatment failures become a major public health problem.

GISP monitored emerging fluoroquinolone-resistant *N. gonorrhoeae* (QRNG) in the United States during the 1990s and 2000s. During this period, fluoroquinolones were widely used for treatment of gonorrhea because they were safe, effective, inexpensive, and available in oral forms. Gonococcal fluoroquinolone resistance, caused by the acquisition of *parC* and *gyrA* mutations that alter binding sites on enzymes DNA gyrase and topoisomerase IV, had emerged in East Asia during the 1990s and was observed sporadically in the United States by GISP. In the early 2000s, QRNG emerged in the United States, spreading initially in Hawaii and California (Figure 1). Men who have sex with men (MSM) were and remain disproportionately affected by QRNG (Figure 2). By 2007, the prevalence of QRNG was >5% among GISP isolates collected throughout the United States, prompting CDC to no longer recommend the use of fluoroquinolones for gonorrhea treatment (3) Spectinomycin, an alternative treatment, had not been available in the United States since 2006, so cephalosporins (such as cefixime and ceftriaxone) were the only remaining antimicrobials recommended for treatment of gonococcal infections.

Cephalosporins remained the foundation of gonorrhea treatment in the 2010 CDC STD treatment guidelines (4). These updated guidelines increased the recommended dosage of ceftriaxone to 250 mg and included broadened recommendations for combination therapy: a cephalosporin, preferably ceftriaxone 250 mg as a single intramuscular dose, should be administered with a second antimicrobial. Combination therapy treats frequently co-occurring pathogens (e.g., *Chlamydia trachomatis*) and might hinder the spread of cephalosporin antimicrobial resistance.

Unsuccessful treatment of gonorrhea with oral cephalosporins, such as cefixime, was identified in East Asia, beginning in the early 2000s, and in Europe within the past few years. Ceftriaxone-resistant isolates have been identified in Japan (2009), France (2010), and Spain (2011) (5–7). GISP now provides growing evidence that cephalosporin resistance might be emerging in the United States. Cefixime minimum inhibitory concentrations (MICs) recently increased, suggesting that the effectiveness of cefixime might be threatened. The percentage of isolates with elevated cefixime MICs (≥0.25 *μ*g/mL) increased from 0.1% in 2006 to 1.4% in 2011 (Figure 3). The increases were most pronounced in isolates collected from men in the western United States and from MSM, the region and population in which QRNG first emerged (8). The acquisition of a mosaic *penA* gene encoding a remodeled penicillin binding protein (PBP2) and overproduction of an efflux pump in *N. gonorrhoeae* appears responsible, at least in part, for reduced susceptibility to cephalosporins.

The development and spread of cephalosporin resistance in *N. gonorrhoeae,* particularly ceftriaxone resistance, would greatly complicate treatment of gonorrhea. Previously recommended antimicrobials likely cannot again be routinely prescribed for empiric gonorrhea treatment. *N. gonorrhoeae* maintains previously acquired antimicrobial resistance phenotypes, even if the antimicrobial is no longer used for treatment. In 2011, 11.8% of isolates in GISP were penicillin-resistant, 22.7% were tetracycline-resistant, and 13.3% were fluoroquinolone-resistant (1). Unlike resistance mutations in many other bacteria, resistance mutations in *N. gonorrhoeae* might actually improve the survival of resistant strains, even in the absence of antimicrobials (9).

New antimicrobial treatment options are needed. However, the number of new systemic antimicrobials approved each year by the Food and Drug Administration has fallen steadily during the past 30 years (10). At this time, only one new antimicrobial is undergoing clinical study (NCT01591447) as a potential treatment for gonorrhea. The National Institute for Allergy and Infectious Diseases, in collaboration with CDC, is conducting a clinical trial (NCT00926796) to study the efficacy of two combinations of existing antimicrobials. The National Institutes of Health currently supports 137 basic science research grants on gonorrhea, including translational research to identify targets for antimicrobial development. Many candidate molecules must be evaluated to find a few that are safe and effective. Antimicrobial drug development is needed now, particularly because the development process for new drugs can take more than a decade.

Challenges in detecting and responding to the emergence of multidrug-resistant gonorrhea also exist. Rapid detection of resistant infections is facilitated by local antimicrobial susceptibility testing, which at this time requires live organisms isolated by culture. However, as the use of nucleic acid amplification tests (NAATs) has expanded, the number of *N. gonorrhoeae* cultures performed by public health laboratories has decreased rapidly (11,12), and the capacity of U.S. laboratories to perform culture for *N. gonorrhoeae* has declined. In addition, many local and state STD programs have experienced reductions in funding and infrastructure in recent years (13), which might hamper the ability of these programs to detect resistant infections and ensure that patients and partners are treated effectively.

## What Public Health Agencies and Partners Can Do

Several steps taken now might delay the emergence of cephalosporin-resistant strains, mitigate the public health consequences of expanded resistance, and prevent a return to the era of untreatable gonorrhea. Local and state STD control programs are encouraged to use local surveillance data to prioritize high-prevalence areas and populations for enhanced primary prevention, screening, or partner services. Clinicians can help prevent sequelae and spread of gonorrhea by eliciting sexual histories from their patients, screening sexually active MSM and high-risk sexually active women for gonorrhea at least annually at exposed anatomic sites, and treating appropriately (4). Clinicians also can counsel sexually active adults, particularly those living in high prevalence areas, to engage in mutually monogamous partnerships with uninfected partners and to consistently and correctly use latex condoms, which can reduce transmission.

Ensuring effective treatment is critical. Based on surveillance trends, CDC recently updated its treatment recommendations: gonorrhea at any anatomic site should be treated with a single 250 mg intramuscular dose of ceftriaxone plus either 1 g of azithromycin as a single oral dose or 100 mg of doxycycline orally twice daily for 7 days (8). If this recommended regimen cannot be used, two alternative treatment options exist for urogenital or rectal gonorrhea: 1) if ceftriaxone is not available, clinicians can consider cefixime 400 mg as a single oral dose and either azithromycin 1 g as a single oral dose or doxycycline 100 mg orally twice daily for 7 days, or 2) if the patient is cephalosporin-allergic, clinicians can consider azithromycin 2 g as a single oral dose. If either of these two alternative regimens is prescribed, the patient should return in 1 week for a test of cure. CDC will continue to update treatment recommendations based on surveillance data and clinical research.

In the United States, GISP is the foundation of gonococcal antimicrobial susceptibility surveillance and has successfully identified important shifts in antimicrobial susceptibility. GISP’s effectiveness can be complemented through enhanced surveillance by local and state health departments. Clinicians can strengthen surveillance by maintaining vigilance for treatment failures, collecting isolates for susceptibility testing from such patients, and promptly notifying the local public health STD program. Local public health laboratories can contribute by maintaining or rebuilding capacity to perform culture for *N. gonorrhoeae* or partnering with laboratories that can. Laboratories that conduct gonococcal antimicrobial susceptibility testing are requested to promptly notify the ordering clinician and local STD control program of isolates with elevated cephalosporin MICs (cefixime MIC ≥0.25 *μ*g/mL or ceftriaxone MIC ≥0.125 *μ*g/mL). Local and state health departments are encouraged to promptly notify CDC of suspected treatment failures or isolates with elevated cephalosporin MICs.

Local and state STD control programs are encouraged to develop local response plans. When a suspected cephalosporin-resistant infection is detected, local public health authorities should interview the patient and ensure adequate treatment and ensure that all recent partners are evaluated and treated appropriately. Working case definitions and more detailed guidance can be found in CDC’s recently released cephalosporin-resistant *N. gonorrhoeae* public health response plan (14).

Within several years, molecular assays for detecting genetic mutations associated with resistance might be available and could enhance surveillance and clinical management. However, molecular assays will not supplant culture-based antimicrobial susceptibility testing for surveillance, which still will be needed to detect novel resistance phenotypes and genotypes. Although a gonococcal vaccine remains an elusive goal, efforts to develop a vaccine are continuing.

Based on past and current data, *N. gonorrhoeae* will continue to acquire antimicrobial resistance. However, experience and current data suggest that public health actions outlined in this report provide the best chance of averting the unfavorable outcome of multidrug-resistant gonorrhea, greater disease burden, heightened risk for sequelae, and greater health-care costs.

## Figures and Tables

**FIGURE 1:**
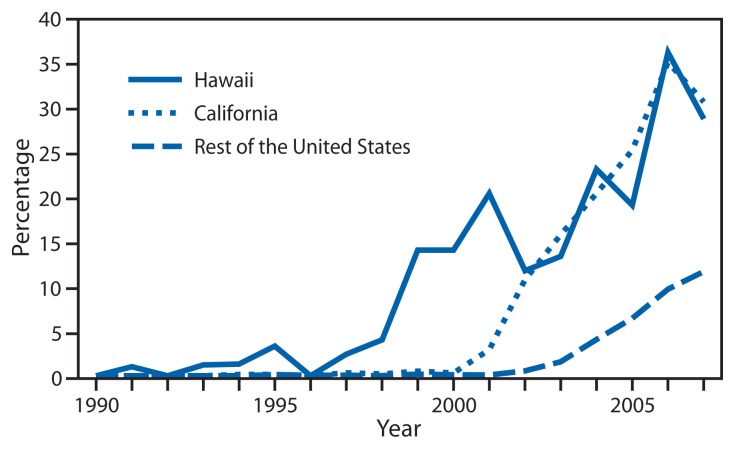
Prevalence of ciprofloxacin resistance* in urethral *Neisseria gonorrhoeae* isolates collected from men in the United States, by location — Gonococcal Isolate Surveillance Project, 1990–2007 * Defined as minimum inhibitory concentrations ≥1 *μ*g/mL.

**FIGURE 2:**
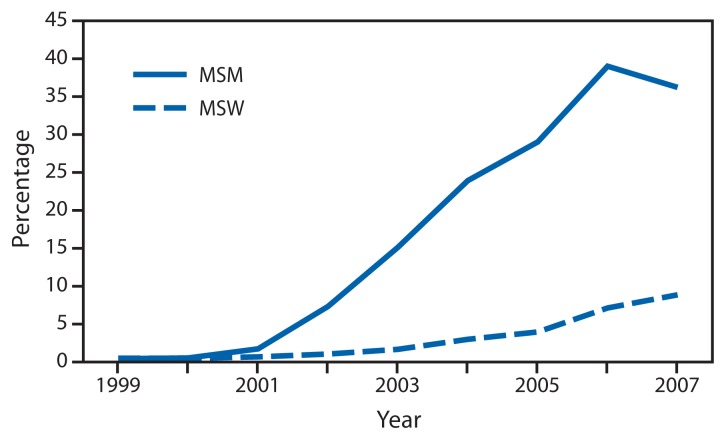
Prevalence of ciprofloxacin resistance* in urethral *Neisseria gonorrhoeae* isolates collected from men in the United States, by gender of sex partner — Gonococcal Isolate Surveillance Project, 1999–2007 **Abbreviations:** MSM = men who have sex with men; MSW = men who have sex exclusively with women. * Defined as minimum inhibitory concentrations ≥1 *μ*g/mL.

**FIGURE 3:**
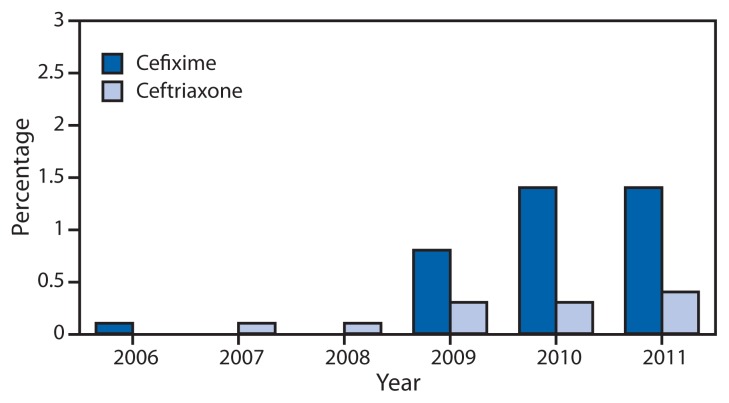
Percentage of urethral *Neisseria gonorrhoeae* isolates with elevated cefixime minimum inhibitory concentrations (MICs) and elevated ceftriaxone MICs* — Gonococcal Isolate Surveillance Project, 2006–2011^†^ * Elevated cefixime MICs defined as ≥0.25 *μ*g/mL; elevated ceftriaxone MICs defined as ≥0.125 *μ*g/mL. ^†^ Isolates not tested for cefixime susceptibility in 2007 and 2008.

## Footnotes

* In GISP, *N. gonorrhoeae* isolates are collected from the first 25 men with urethral gonorrhea attending STD clinics each month in approximately 28 cities in the United States. At regional laboratories, the susceptibilities of these isolates to penicillin, tetracycline, spectinomycin, ciprofloxacin, ceftriaxone, cefixime, and azithromycin are determined by agar dilution. Minimum inhibitory concentrations are measured, and values are interpreted according to criteria recommended by the Clinical and Laboratory Standards Institute. The GISP protocol, which describes the methodology, is available at http://www.cdc.gov/std/gisp/gisp-protocol07-15-2010.pdf. Additional information is available at http://www.cdc.gov/std/gisp/default.htm.

## References

[1] CDC (2012). Sexually transmitted disease surveillance 2011.

[2] Newman LM, Berman SM (2008). Epidemiology of STD disparities in African American communities. Sex Transm Dis.

[3] CDC (2007). Update to CDC’s sexually transmitted diseases treatment guidelines, 2006: fluoroquinolones no longer recommended for treatment of gonococcal infections. MMWR.

[4] CDC (2010). Sexually transmitted diseases treatment guidelines, 2010. MMWR.

[5] Ohnishi M, Golparian D, Shimuta K (2011). Is *Neisseria gonorrhoeae* initiating a future era of untreatable gonorrhea?: detailed characterization of the first strain with high-level resistance to ceftriaxone. Antimicrob Agents Chemother.

[6] Unemo M, Golparian D, Nicholas R, Ohnishi M, Gallay A, Sednaoui P (2012). High-level cefixime- and ceftriaxone-resistant *Neisseria gonorrhoeae* in France: novel *penA* mosaic allele in a successful international clone causes treatment failure. Antimicrob Agents Chemother.

[7] Cámara J, Serra J, Ayats J (2012). Molecular characterization of two high-level ceftriaxone-resistant *Neisseria gonorrhoeae* isolates detected in Catalonia, Spain. J Antimicrob Chemother.

[8] CDC (2012). Update to CDC’s sexually transmitted diseases treatment 2010 guidelines: oral cephalosporins no longer a recommended treatment for gonococcal infections. MMWR.

[9] Kunz AN, Begum AA, D’Ambrozio JA (2012). Impact of fluoroquinolone resistance mutations on gonococcal fitness and in vivo selection for compensatory mutations. J Infect Dis.

[10] Spellberg B, Guidos R, Gilbert D (2008). The epidemic of antibiotic-resistant infections: a call to action for the medical community from the Infectious Diseases Society of America. Clin Infect Dis.

[11] Dicker LW, Mosure DJ, Steece R, Stone KM (2004). Laboratory tests used in US public health laboratories for sexually transmitted diseases, 2000. Sex Transm Dis.

[12] CDC (2011). Volume and type of laboratory testing methods for sexually transmitted diseases in public health laboratories, 2007.

[13] Wong W, Miller S, Rabins C STD program capacity and preparedness in the United States: results of a national survey, 2009 (Abstract 22048).

[14] CDC (2012). Cephalosporin-resistant *Neisseria gonorrhoeae* public health response plan.

